# Effects of Sacha Inchi (*Plukenetia volubilis* L.) Oil Supplementation on Hyperglycaemia, Hypertension and Hyperlipidaemia (3Hs) Patients: A Preliminary Human Trial

**DOI:** 10.1007/s11130-025-01309-8

**Published:** 2025-02-25

**Authors:** Nur Anis Raihana Mhd Rodzi, Mastura Mohd Sopian, Lai Kuan Lee

**Affiliations:** 1 Present Address: Food Technology Program, School of Industrial Technology, Universiti Sains Malaysia, Gelugor, Pulau Pinang 11800 Malaysia; 2 Clinical Medicine Department, Universiti Sains Malaysia Bertam Medical Centre, Kepala Batas, Pulau Pinang 13200 Malaysia

**Keywords:** Complementary medicine, Sacha inchi, Polyunsaturated fatty acids, Hyperglycaemia, Hyperlipidaemia, Hypertension

## Abstract

Sacha Inchi oil (SIO), derived from seeds of *Plukenetia volubilis L.*, is a novel plant source of unsaturated fatty acids, highly regarded for its health benefits. The rich fractions of polyunsaturated fatty acids have shown potential in improving or reducing the impact of hyperglycaemia, hypertension and hyperlipidaemia (3Hs) for both in vivo and preliminary human studies. This study aimed to investigate the effects of SIO supplementation on glycaemic, lipid and blood pressures outcomes of patients with 3Hs. A randomised, double-blind, placebo-controlled human clinical trial was conducted to investigate the effects of SIO supplementation in fifty-four (*n* = 54) 3Hs patients. Assessments of glycaemic control, blood pressure and lipid profiles were conducted and compared at baseline and week-12 after 3Hs patients were randomised into SIO group (*n* = 27) (received 1000 mg of SIO soft gel per day) or the placebo group (*n* = 27) (received 1000 mg of corn oil), with prescribed medications. The compliancy and tolerability of supplementation were assessed concurrently. The SIO group demonstrated lower systolic (-8.6 mmHg, *p* = 0.004) and diastolic blood pressure (BP) (-7.0 mmHg, *p* = 0.004), total cholesterol (TC) (-0.6 mmol/L, *p* = 0.024), low density lipoprotein-cholesterol (LDL-C) (-0.3 mmol/L, *p* = 0.036) and high density lipoprotein-cholesterol (HDL-C) (*p* = 0.048) but no effect on glycaemic markers. No clinical adverse events were reported, and the side effects of SIO supplementation were self-limiting and minimal. SIO supplementation showed the potential to alleviate BPs, reduced both TC and LDL-C, and improved HDL-C levels. SIO may offer a simple, novel complementary medicine for the management of 3Hs.

**Supplementary Information:**

The online version contains supplementary material available at 10.1007/s11130-025-01309-8.

## Introduction

Hyperglycaemia, characterised by elevated blood glucose due to insulin resistance (IR) and β-cell dysfunction, is a key feature of type 2 diabetes mellitus (T2DM) [1]. Hypertension or elevated blood pressure (BP) is another component of metabolic syndrome, both of which can lead to peripheral vascular disease, kidney damage, and visual impairment [2–4]. Hyperlipidaemia, defined by elevated fasting cholesterol, is often associated with increased triglycerides (TG) [5, 6]. These conditions may arise from various comorbidities or lifestyle factors and their presence independently increases cardiovascular risk [2, 6].

*Sacha* Inchi oil (SIO) is gaining popularity owing to its high content of omega-3 (Ω-3) fatty acids, such as alpha linolenic acid (ALA) [7]. ALA activates the receptor responsible for insulin sensitisation and the enhancement of glucose metabolism (PPARy) [8]. Clinical trials have suggested significant reductions in BP following plant-based oil [9]. Omega-3 fatty acids exert lowering effects in hyperlipidaemic patients by decreasing hepatic lipogenesis, increasing β-oxidation, inhibiting key enzymes involved in hepatic TG synthesis, and increasing the expression of lipoprotein lipase, leading to increased TG removal from circulating very low density lipoprotein (VLDL) and chylomicron particles [10, 11].

A pioneer, double-blind, randomised, crossover trial found that 15 ml SIO supplementation into high-fat breakfast among the metabolically unhealthy individuals reduced blood glucose levels and increased Sirtuin (SIRT-1) expression, a protein that enhances insulin secretion and activates insulin pathways [12]. Contradictorily, a 4 months trial (10 or 15 ml SIO supplementation given to healthy adults) did not exhibit any significant change in regular and fasting blood glucose (FBG) levels [13]. Another trial conducted among the hypercholesterolaemic patients reported significant reductions in lipid profiles after 4 months of SIO supplementation [14]. In animal study, daily ingestion of 400 mg/kg Sacha Inchi diet was capable to reduce BP and improved oxidative damage in rats [15]. In an earlier study, 0.5 ml/kgBW of SIO in Holtzman rats improved liver function, lowered cholesterol level and TG, increased HDL-C, and elevated insulin levels [16].

To the best of our knowledge, no research has examined the effects of SIO supplementation as a complimentary regimen for 3Hs patients. This study aimed to assess the effects of SIO supplementation on primary 3H indicators, including FBG, glycated haemoglobin (HbA1c), fasting serum insulin, insulin resistance (HOMA-IR), systolic and diastolic BP, and lipid profiles. The safety, tolerability, and adherence to the supplementation were also evaluated.

## Materials and Methods

The Materials and Methods section is presented as supplementary material.

## Results and Discussions

### Subjects

This was the first randomised human clinical trial in the Southeast Asia to evaluate the effects of SIO supplementation among patients with 3Hs. Fifty-four 3Hs patients were randomly categorised into the SIO or placebo group (*n* = 27 *per* group). At baseline, we observed no significant difference in any of the socio-demographic characteristics, nutritional status assessments, and the plasma fatty acid composition. The SIO group, however, performed more frequent physical activity (*p* < 0.05), while a greater proportion of patients in the placebo group were smokers (*p* < 0.0001) (Table S5).

### Effects of SIO Versus Placebo Supplementation on Glycaemic Markers

The baseline and post intervention glycaemic marker levels are shown in Table 1. No significant changes in FBG, HbA1c, FSI or HOMA-IR were detected either in the SIO or placebo group. Hence, our study suggested that SIO supplementation did not affect glycaemic marker levels, in line with Foster et al. [17]. However, Alayón et al. [12] showed an attenuation effect on the elevation of blood glucose and a marked increase in the expression of SIRT-1 in the SIO group. SIRT1 is a vital protein in the human body that stimulates glucose-dependent insulin secretion [12]. According to a recent meta-analysis, plant-derived omega-3 PUFA supplementation ameliorates insulin resistance [18]. It is believed that omega-3 PUFAs reduce FBG levels by enhancing the sensitivity of insulin signals induced by G-coupling receptors of glucagon-like peptide 1 (GLP-1) and regulating the signalling pathways involved in insulin production [19]. The discrepant findings suggest the need for a larger sample size for human trials, and future studies are needed to unravel the complex interrelationship between SIO consumption and glycaemic control.

**Table 1:** Effects of SIO versus placebo on glycaemic markers, blood pressure and lipid profile

Variable		Baseline	12-week	*p* ^1^	*p* ^2^	F	Power	ηp^2^
FBG (mmol/L)	SIO	8.6 ± 3.1	8.7 ± 2.8	0.781	0.416	0.679	0.126	0.020
	Placebo	7.3 ± 1.8	7.8 ± 2.3					
HbA1c (%)	SIO	7.6 ± 2.1	7.5 ± 1.5	0.869	0.151	2.159	0.298	0.058
	Placebo	7.1 ± 1.2	7.3 ± 1.2					
FSI (microIU/mL)	SIO	15.8 ± 0.6	15.8 ± 0.5	0.388	0.389	0.762	0.136	0.021
	Placebo	15.8 ± 0.8	15.7 ± 0.7					
HOMA-IR	SIO	6.0 ± 2.1	6.1 ± 2.0	0.791	0.347	0.908	0.153	0.025
	Placebo	5.1 ± 1.2	5.4 ± 1.7					
SBP (mmHg)	SIO	143.9 ± 2.6	135.3 ± 2.5	0.003**	0.004**	8.962	0.835	0.152
	Placebo	143.9 ± 2.5	144.6 ± 2.5					
DBP (mmHg)	SIO	83.9 ± 1.4	76.9 ± 1.5	< 0.0001**	0.004**	9.271	0.848	0.156
	Placebo	82.6 ± 1.4	81.9 ± 1.5					
TC (mmol/L)	SIO	5.1 ± 0.6	4.5 ± 0.6	< 0.0001***	0.024*	5.607	0.633	0.633
	Placebo	4.6 ± 0.8	4.6 ± 1.3					
LDL-C (mmol/L)	SIO	2.5 ± 0.9	2.2 ± 0.7	0.008**	0.036*	4.771	0.565	0.123
	Placebo	2.5 ± 0.8	2.7 ± 1.2					
HDL-C (mmol/L)	SIO	1.2 ± 0.2	1.2 ± 0.2	0.975	0.048*	4.114	0.512	0.076
	Placebo	1.2 ± 0.1	1.1 ± 0.1					
TG (mmol/L)	SIO	1.7 ± 0.7	1.7 ± 0.6	0.656	0.876	0.025	0.053	0.000
	Placebo	1.6 ± 0.5	1.7 ± 0.5					

Data are presented as mean ± standard deviation (SD)

*p < 0.05, **p < 0.01, ***p < 0.0001

*p*^*1*^ = *p* values generated using Independent Student’s t-test; *p*^*2*^ *= p*-values are reported based on repeated measures ANCOVA after adjustment for age, physical activity, smoking status and prescribed medication

FBG = fasting blood glucose; HbA1c = glycated haemoglobin A1c; FSI = fasting serum insulin; HOMA-IR = homeostatic model assessment of insulin resistance, SBP = systolic blood pressure; DBP = diastolic blood pressure, TC = total cholesterol; LDL-C = low-density lipoprotein cholesterol; HDL-C = high-density lipoprotein cholesterol; TG = triglyceride

### Effects of SIO Versus Placebo Supplementation on Systolic and Diastolic BPs

Compared with the placebo group, 3Hs patients who received SIO had significantly lower systolic (-7.8 ± 0.6 vs. -0.04 ± 0.3 mmHg; *p* = 0.004) and diastolic BP (-7.1 ± 1.1 vs. -0.6 ± 1.1 mmHg; *p* = 0.004) (Table 1). Previous study reported that 10 or 15 mL of SIO emulsion reduced arterial BPs [11]. BP and vascular function are regulated through the reversible nitric oxide-sensitive guanylate cyclase (NOsGC) pathway [20]. The pathway that produces endothelial nitric oxide (eNOS) is the primary source of blood NO [21]. eNOS knockout leads to hypertension and vascular dysfunction [22]. Inadvertently, SIO exhibits radical scavenging activities against anionic, hydroxyl, ABTS and DPPH radicals that increase the pinocytosis of cells and increases NO production [23]. We suggest that BP reduction via PUFA consumption yields additional clinical benefits, as the n-6:n-3 ratio in our SIO supplement was 1:1.25, which is considered to be an almost ideal ratio. A balance n-6:n-3 fatty acid ratio is crucial for reducing the risk of coronary heart disease [24].

### Effects of SIO Versus Placebo Supplementation on Lipid Profiles

Significant improvements in TC (-0.5 ± 0.7 vs. 0.05 ± 0.6 mmol/L; *p* = 0.024), LDL-C (-0.3 ± 0.7 vs. 0.2 ± 0.6 mmol/L; *p* = 0.036), and HDL-C (0.001 ± 0.1 vs. -0.043 ± 0.0 mmol/L; *p* = 0.048) were observed in the SIO group after 12 weeks intervention (Table 1). The serum TG level remained unchange. Four months of consumption of 5 or 10 mL of SIO emulsion significantly reduced TC, LDL-C, non-HDL cholesterol, TG, VLDL and NEFAs in 24 hypercholesterolemic patients [25]. An organoleptic study reported that SIO-supplemented high-fat diet attenuated the increase in TC, in addition to a reduction in interleukin-6 [26].

ALA is known to modulate lipid metabolism, improve lipid homeostasis, increase fatty acid β-oxidation through the upregulation of peroxisome-activated receptor-ɑ and the downregulation of sterol regulatory element-binding protein-1 [27]. Long chain omega-3 PUFAs, which are highly abundant in SIO, have potential health benefits for the modulation of balanced lipid profiles [28]. Earlier literature found that SIO consumption mitigated the increase in lipopolysaccharide (LPS) entry into circulation, thus suppressing the correlation between LPS and TG through the inhibitory effect of PUFAs, even in the presence of high TG levels [29, 30]. In addition, the effects of omega-3 PUFAs on lipolysis can be modulated by perilipin and/or hormone-sensitive lipase (HSL). Perilipin recovers intracellular lipidic particles in adipocytes, increases the access of HSL to adipocytes, and hydrolyses fatty droplets, leading to increased lipolysis. The lipoprotein lipase enzyme releases fatty acids through the hydrolysis of chylomicron and VLDL-triacylglycerol, reducing their level in the blood, thus resulting in a lipid-lowering effect [23].

The current SIO supplement contributed approximately 88.8 mg of omega-9 fatty acid per day. Oleic acid is associated with cardiovascular insulin resistance, improved endothelial dysfunction in response to proinflammatory signals, and reduced proliferation and apoptosis in vascular smooth muscle cells (VSMCs) [31, 32]. As such, we propose that oleic acid may partly involve in instability and underlying complications in the context of vascular smooth muscle remodelling. However, the detail pathway is yet to be fully understood.

### Compliance, Tolerability and Adherence

All participants completed the trial. Most of the patients were well tolerated to the supplementation regimen. The main complaints were mild nausea (*n* = 4) and mild gastrointestinal discomfort (*n* = 2) during the first week of consumption. Adherence to the trial was 97% for the SIO group and 95% for the placebo group, respectively.

The levels of ALA, linoleic acid (LA), oleic acid, total omega-3 PUFAs and total omega-6 PUFAs were significantly increased (all *p* < 0.05) after intervention with SIO (data not shown). In parallel, the plasma LA (*p* < 0.05) and total omega-6 PUFAs were increased significantly (*p* < 0.05) in the placebo group.

## Strengths and Limitations

This was a preliminary trial to explore the role of SIO supplementation for the amelioration of hyperglycaemia, hypertension and hyperlipidaemia. Daily supplementation with 1000 mg of SIO for 12 weeks significantly improved BP and lipid profiles in patients with 3Hs. Both trial groups reported high supplement adherence throughout the study. As such, nutritional supplement interventions could be the primary strategies for ameliorating hypertension and hyperlipidaemia. Furthermore, each patient underwent direct screening and recruitment procedures. By enrolling patients with similar characteristics and conditions, the variability within the study population is minimised. Nonetheless, our study has several limitations. These limitations include the relatively small sample size, which commonly arises due to the use of stringent inclusion and exclusion criteria in the trial. We suggest that a longer study period may be recommended for future studies to assess more accurate intervention outcomes. Furthermore, it would be beneficial to measure the NO levels in 3Hs patients.

## Conclusion

This preliminary study demonstrated encouraging results of SIO supplementation on BP and lipid profile management in individuals with 3Hs. SIO supplementation has the potential to alleviate BPs, while reducing TC and LDL-C and improving HDL-C. This trial may contribute to the dissemination of reliable findings that may support the recommendation of the novel SIO as complementary medicine. Future studies with larger sample sizes, longer intervention periods and modified SIO compositions should be planned before conclusive recommendations can be made.

## Electronic Supplementary Material

Below is the link to the electronic supplementary material.

Supplementary Material 1

Supplementary Material 2

## Data Availability

Data cannot be shared openly to protect the patients’ privacy.

## References

[1] Clinical Practice Guidelines (2020) Management of type 2 diabetes Mellitus, Sixth Edition. Ministry of Health, Malaysia., Putrajaya

[2] Clinical Practice Guidelines: Management of Hypertension 5th Edition (2018) Ministry of Health Malaysia. 160

[3] Singh S, Shankar R, Singh GP (2017) Prevalence and Associated Risk factors of hypertension: a cross-sectional study in Urban Varanasi. Int J Hypertens 2017:1–10. 10.1155/2017/5491838 10.1155/2017/5491838 PMC5733954 29348933

[4] Farhat A, Al-Hajje A, Rachidi S et al (2016) Risk factors and quality of life of dyslipidemic patients in Lebanon: a cross-sectional study. J Epidemiol Global Health 6(4):315. 10.1016/j.jegh.2016.10.001 10.1016/j.jegh.2016.10.001 PMC7320458 27842211

[5] 5th Edition of Clinical Practice Guidelines: Management of Dyslipidaemia. (2017)

[6] Magliano D, Boyko EJ (2021) IDF diabetes atlas. 10th edition. Brussels: International Diabetes Federation; https://www.ncbi.nlm.nih.gov/books/NBK581934/

[7] Sánchez EG, Hernández-Ledesma B, Gutiérrez LF (2021) Sacha Inchi Oil Press-cake: physicochemical characteristics, Food-related applications and biological activity. Food Reviews Int 1–12. 10.1080/87559129.2021.1900231

[8] Wang JF, Zhang HM, Li YY et al (2019) A combination of omega-3 and plant sterols regulate glucose and lipid metabolism in individuals with impaired glucose regulation: a randomized and controlled clinical trial. Lipids Health Dis 18(106):9. 10.1186/s12944-019-1048-x 31043161 10.1186/s12944-019-1048-x PMC6495649

[9] Ursoniu S, Sahebkar A, Andrica F et al (2016) Effects of flaxseed supplements on blood pressure: a systematic review and meta-analysis of controlled clinical trial. Clin Nutr 35(3):615–625. 10.1016/j.clnu.2015.05.012 26071633 10.1016/j.clnu.2015.05.012

[10] Esherick J, Slater E, David J Current Practice Guidelines in Primary Care 2020

[11] Breda J, Wickramasinghe K, Peters DH et al (2019) One size does not fit all: implementation of interventions for non-communicable diseases. BMJ l6434. 10.1136/bmj.l6434 10.1136/bmj.l6434 31810904

[12] Alayón AN, Ortega Avila JG, Echeverri Jiménez I (2018) Carbohydrate metabolism and gene expression of sirtuin 1 in healthy subjects after Sacha Inchi oil supplementation: a randomized trial. Food Funct 9(3):1570–1577. 10.1039/c7fo01956d 29437170 10.1039/c7fo01956d

[13] Gonzales GF, Gonzales C (2014) A randomized, double-blind placebo-controlled study on acceptability, safety and efficacy of oral administration of Sacha Inchi oil (Plukenetia Volubilis L.) in adult human subjects. Food Chem Toxicol 65:168–176. 10.1016/j.fct.2013.12.039 24389453 10.1016/j.fct.2013.12.039

[14] Gonzales GF, Gonzales C, Villegas L (2014) Exposure of fatty acids after a single oral administration of Sacha Inchi (*Plukenetia Volubilis* L.) and sunflower oil in human adult subjects. Toxicol Mech Methods 24(1):60–69. 10.3109/15376516.2013.850566 24090048 10.3109/15376516.2013.850566

[15] Wu X, Song M, Qiu P et al (2018) A metabolite of nobiletin, 4′-demethylnobiletin and atorvastatin synergistically inhibits human colon cancer cell growth by inducing G0/G1 cell cycle arrest and apoptosis. Food Funct 9(1):87–95. 10.1039/c7fo01155e 29063088 10.1039/c7fo01155e PMC6375479

[16] Gorriti A, Arroyo J, Quispe F et al (2010) (Plukenetia Volubilis L.) Y LINAZA (Linum usitatissimum L). Rev Peru Med Exp Salud Publica 27(3):9. 10.1590/S1726-46342011000400009 10.1590/s1726-46342010000300007 21152727

[17] Foster M, Petocz P, Caterson ID et al (2013) Effects of zinc and α-linolenic acid supplementation on glycemia and lipidemia in women with type 2 diabetes mellitus: a randomized, double-blind, placebo-controlled trial. J Diab Res Clin Met 2(1):3. 10.7243/2050-0866-2-3

[18] Zhou J, Zuo W, Tan Y et al (2023) Effects of n-3 polyunsaturated fatty acid on metabolic status in women with polycystic ovary syndrome: a meta-analysis of randomized controlled trials. J Ovarian Res 16(1):54. 10.1186/s13048-023-01130-4 36932420 10.1186/s13048-023-01130-4 PMC10022207

[19] Savini I, Catani M, Evangelista D et al (2013) Obesity-Associated oxidative stress: strategies finalized to Improve Redox State. IJMS 14(5):10497–10538. 10.3390/ijms140510497 23698776 10.3390/ijms140510497 PMC3676851

[20] Ma J, Li Y, Yang X et al (2023) Signalling pathways in vascular function and hypertension: molecular mechanisms and therapeutic interventions. Sig Transduct Target Ther 8(1):168. 10.1038/s41392-023-01430-7 10.1038/s41392-023-01430-7 PMC10119183 37080965

[21] Li Q, Youn JY, Cai H (2015) Mechanisms and consequences of endothelial nitric oxide synthase dysfunction in hypertension. J Hypertens 33(6):1128–1136. 10.1097/HJH.0000000000000587 25882860 10.1097/HJH.0000000000000587 PMC4816601

[22] Gallo G, Volpe M, Savoia C (2022) Endothelial dysfunction in hypertension: current concepts and clinical implications. Front Med 8:798958. 10.3389/fmed.2021.798958 10.3389/fmed.2021.798958 PMC8811286 35127755

[23] Li P, Huang J, Xiao N et al (2018) Sacha Inchi oil alleviates gut microbiota dysbiosis and improves hepatic lipid dysmetabolism in high-fat diet-fed rats. Food Funct 9(1):87–95. 10.1039/d0fo01178a 32648886 10.1039/d0fo01178a

[24] Patel A, Desai SS, Mane VK et al (2022) Futuristic food fortification with a balanced ratio of dietary ω-3/ω-6 omega fatty acids for the prevention of lifestyle diseases. Trends Food Sci Technol 120:140–153. 10.1016/j.tifs.2022.01.006

[25] Garmendia F, Pando R, Ronceros G (2014) Efecto Del aceite de sacha inchi (Plukenetia volúbilis L) Sobre El Perfil lipídico en pacientes con hiperlipoproteinemia. Rev Peru Med Exp Salud Publica 28(4). 10.1590/S1726-46342011000400009 22241259

[26] Alayón AN, Ortega JG, Echeverri JI (2019) Metabolic status is related to the effects of adding of Sacha Inchi (*Plukenetia Volubilis L*.) oil on postprandial inflammation and lipid profile: Randomized, crossover clinical trial. J Food Biochem 43(2):e12703. 10.1111/jfbc.12703 31353666 10.1111/jfbc.12703

[27] Saleh-Ghadimi S, Kheirouri S, Golmohammadi A et al (2019) Effect of flaxseed oil supplementation on anthropometric and metabolic indices in patients with coronary artery disease: a double-blinded randomized controlled trial. J Cardiovasc Thorac Res 11(2):152–160. 10.15171/jcvtr.2019.26 31384411 10.15171/jcvtr.2019.26 PMC6669420

[28] Chang E, Kim C (2019) Natural products and obesity: a focus on the regulation of mitotic clonal expansion during Adipogenesis. Molecules 24(6):1157. 10.3390/molecules24061157 30909556 10.3390/molecules24061157 PMC6471203

[29] Lyte JM, Gabler NK, Hollis JH (2016) Postprandial serum endotoxin in healthy humans is modulated by dietary fat in a randomized, controlled, cross-over study. Lipids Health Dis 15(1):186. 10.1186/s12944-016-0357-6 27816052 10.1186/s12944-016-0357-6 PMC5097840

[30] Kell DB, Pretorius E (2015) On the translocation of bacteria and their lipopolysaccharides between blood and peripheral locations in chronic, inflammatory diseases: the central roles of LPS and LPS-induced cell death. Integr Biology 7(11):1339–1377. 10.1039/c5ib00158g 10.1039/c5ib00158g 26345428

[31] Shetty SS, Kumari NS, Shetty PK (2020) ω-6/ω-3 fatty acid ratio as an essential predictive biomarker in the management of type 2 diabetes mellitus. Nutrition 79–80. 10.1016/j.nut.2020.110968 10.1016/j.nut.2020.110968 32919185

[32] Perdomo L, Beneit N, Otero YF et al (2015) Protective role of oleic acid against cardiovascular insulin resistance and in the early and late cellular atherosclerotic process. Cardiovasc Diabetol 14(1):75. 10.1186/s12933-015-0237-9 26055507 10.1186/s12933-015-0237-9 PMC4475625

