# Supplementary material for: Effects of Sacha Inchi (Plukenetia volubilis L.) Oil Supplementation on Hyperglycaemia, Hypertension and Hyperlipidaemia (3Hs) Patients: A Preliminary Human Trial
Source: Plant Foods Hum Nutr. 2025 Feb 25;80(1):80. doi: 10.1007/s11130-025-01309-8 (PMC11861005; doi:10.1007/s11130-025-01309-8)
Supplement: Supplementary file 1 — Supplementary Material 1 [file 11130_2025_1309_MOESM1_ESM.docx]

**Document Title: Supplementary Materials (Materials & Methods)**

**Journal: Plant Foods for Human Nutrition**

**Article Title: Effects of Sacha Inchi (Plukenetia volubilis L.) oil supplementation on hyperglycaemia, hypertension and hyperlipidaemia (3Hs) patients: A preliminary human trial**

Nur Anis Raihana Mhd Rodzi, PhD^1^, Mastura Mohd Sopian, MMed^2^, Lai Kuan Lee, PhD^1*^

**^1^**Food Technology Program, School of Industrial Technology, Universiti Sains Malaysia, 11800 Gelugor, Pulau Pinang, Malaysia.

**^2^**Clinical Medicine Department, Universiti Sains Malaysia Bertam Medical Centre, 13200 Kepala Batas, Pulau Pinang, Malaysia.

Corresponding author*

Lai Kuan Lee, PhD

Food Technology Program, School of Industrial Technology, Universiti Sains Malaysia, 11800 Gelugor, Pulau Pinang, Malaysia

Email: [l.k.lee@usm.my](mailto:l.k.lee@usm.my)

Phone: +604-6536360

Fax: +604-6536375

**Materials & methods**

***Subjects***

3Hs subjects were recruited were registered 3Hs patients who received standard care at the Day Care Clinic in the Universiti Sains Malaysia Bertam Medical Centre. Table S1 shows the subjects inclusion and exclusion criteria for the trial. Study recruitment and enrolment began on May 1^st^, 2021, and the completion date for the trial was December 31st, 2022. The following formula was adopted as a reference [1]:

Where

Z** is equal to 0.8416 (80% power and 95% confidence interval) and *Z*= 1.96. Assuming a dropout rate of 20%, the required sample size was approximately 50 participants—25 patients in the intervention group and 25 patients in the placebo group.

The trial followed the Declaration of Helsinki and CONSORT guidelines. All participants provided consent, including for publication. The study was approved by the USM Human Research Ethics Committee (JEPEM) (protocol certification number: USM/JEPeM/20120710) and registered with the National Medical Research Register (NMRR) (NMRR-20-3234-56756) and ClinicalTrials.gov (ID: NCT04920825). The link for the registration is accessible at <https://shorturl.at/pPq5b>.

**Table S1.** Inclusion and exclusion criteria for the study population.

| **Inclusion criteria** | **Exclusion criteria** |
| --- | --- |
| **Chronological age 18 years and above** | **Comorbidities** |
| **Male or female** | Liver, kidney and haematological disorders, active gastric/duodenal ulcer, cancer and endo­­crine disorders |
| **3Hs ≥ 6 months and free from any complications** | **Undergoing therapy** |
| HbA1c 6.5-8.5%; BP >140/90 mmHg; TC >5.2 mmol/L; HDL-C <1.0 mmol/L (males), <1.2 mmol/L (females); TG > 1.7 mmol/L, LDL-C >3.4 mmol/L | Hormone replacement therapy, use of steroids, chemotherapy, immunosuppressant or radiotherapy |
| **Pharmacological treatment with standard medication regimen** | **Mentally unstable** |
| Oral anti-diabetics (metformin, sulphonylureas, meglitinides, alpha-glucosidase inhibitors, Thiazolidinediones, DPP-4 Inhibitors, or SGLT2 Inhibitors) and/or injecting agents (GLP-1 Receptor Agonists, anti- hypertensive agents (diuretics, beta blockers, calcium channel blockers, ACE inhibitors, angiotensin inhibitor blockers and direct renin inhibitors) and lipid modifying agents (statins, fibrates, PCSK 9 inhibitors, anion exchange resins, niacin and cholesterol absorption inhibitors) | Psychiatric disease/mental retardation |
| **Not involved in other intervention program** | **Pregnant & lactating mothers** |

HbA1c, glycated haemoglobin A1c; TC, total cholesterol; HDL-C, high density lipoprotein cholesterol; LDL-C, low density lipoprotein cholesterol; TG, triglyceride**;** DPP-4, Dipeptidyl Peptidase-4 Inhibitors**;** SGLT2, Sodium-glucose Cotransporter 2; GLP-1, Glucagon-like Peptide-1

***Study schedule and grouping***

A randomised, double-blind, placebo controlled design was used. Five levels of study schedule were adopted in this trial: (i) screening, recruitment, and informed consent signing; (ii) randomisation and blinding; (iii) enrolment visit; (iv) follow-up visits; and (v) post week-12 visit adhering to the CONSORT guidelines. Figure S1 illustrates the trial flowchart. Patients with 3Hs were identified through a registry check and invited to participate using information leaflets and posters. After obtaining informed consent and confirming eligibility, patients were randomly assigned into supplement group (S) or placebo group (P), with 1:1 allocation, using a computed-generated list and permuted block randomisation by the statistician, with allocation concealed in sealed, numbered envelopes. None of investigators nor patients were aware of the allocation until the study was completed. Table S2 outlines the study schedule. Each participant's socio-demographic profile, medical history, clinical outcomes, and safety were assessed. Upon enrolment, participants completed a questionnaire and provided a 20 mL fasting blood sample. Patients were evaluated at weeks 4, 8, and after week 12 to assess safety, tolerability, and compliance with the supplement, with daily intake recorded and monitored weekly. At week 12, clinical and compliance assessments were repeated. Participants received a month's supply of soft gel supplements in pre-labelled bottles and were asked to return the bottles at follow-up visits to track adherence. Participants were asked not to change their diet or physical activity, and any adverse effects were documented.

The supplement group (S) (n = 27) was supplemented with 1000 mg (2 soft gels, 500 mg each) of SIO (equivalent to 461.4 mg omega-3, 367.6 mg omega-6, and 88.8 mg omega-9) for 12 weeks (Table S3). SIO was sponsored by Orient Biotech Sdn. Bhd. The placebo group was given 1000 mg (2 soft gels, 500 mg each) of corn oil for up to 12 weeks. Participants were instructed to consume 1 capsule of soft gel in the morning after breakfast, and 1 capsule of soft gel in the evening after a meal. All patients continued to take their regular medications as prescribed beforehand.


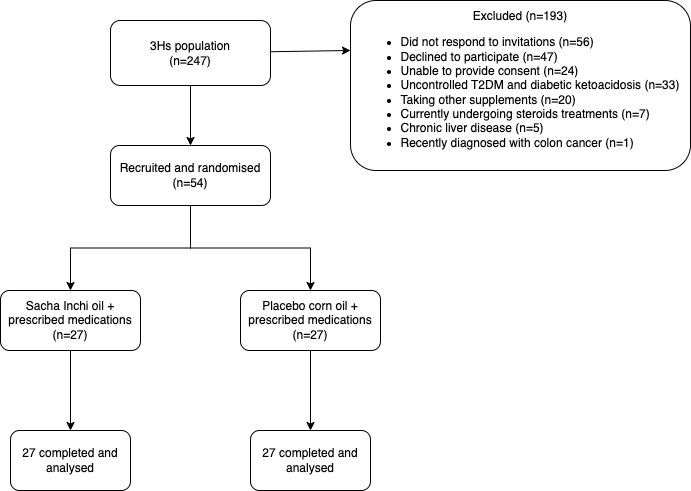
**Fig. S1**. Study design flow chart

**Table S2**. Study schedule of assessments.

| **Study phase** | **Screening stage** | **Baseline** | **Wk-4** | **Wk-8** | **Wk-12** | |
| --- | --- | --- | --- | --- | --- | --- |
| Recruitment | ✓ |  |  |  | |  |
| Intervention |  |  |  |  | |  |
| Socio-demographic |  | ✓ |  |  | |  |
| Medical history |  | ✓ |  |  | |  |
| Clinical data (fasting blood glucose, HbA1c, fasting insulin, HOMA-IR, blood pressure, liver and kidney function) |  | ✓ |  |  | | ✓ |
| Laboratory data (lipid profiles) |  | ✓ |  |  | | ✓ |
| Progression of complications |  | ✓ | ✓ | ✓ | | ✓ |
| Safety & tolerability |  | ✓ | ✓ | ✓ | | ✓ |
| Compliance |  | ✓ | ✓ | ✓ | | ✓ |

✓, required

Medical history included current medical history (symptoms and signs) and previous history.

Safety and tolerability were assessed for gastrointestinal discomfort that may have occurred during the study.

HbA1c: glycated haemoglobin A1c; HOMA-IR: homeostatic model assessment of insulin resistance

**Table S3.** Active ingredients of the Sacha Inchi oil softgel

| **Active Ingredients** | **Composition**  **(500 mg)** | **Composition**  **(1000 mg)** |
| --- | --- | --- |
| Omega-3 PUFAs | 230.7 | 461.1 |
| Omega-6 PUFAs | 183.8 | 367.6 |
| Omega-9 PUFAs | 44.4 | 88.8 |

***Study outcomes and measures***

The patients’ outcome measures were assessed at two time points: at enrolment (baseline) and at the end of the intervention period (post week-12). A fasting blood sample (20 mL of brachial venous blood) with a variation of refrigerated tubes at both time points was collected to measure all outcomes shown in Table S4. The collected samples were centrifuged at 2000 rpm at 4°C for 10 min. Subsequently, the aliquot samples were stored in a -80°C freezer for subsequent analysis. The outcomes for the trial remained throughout the whole study duration. Primary outcomes included glycaemic markers (FBG, HbA1c, FSI, and HOMA-IR), blood pressure, and lipid profiles, all measured using ELISA Kit (Elabscience^®^, United States) and standard equipments. Additional data on socio-demographics, lifestyle, body measurements, and plasma fatty acid composition were recorded and analysed. The plasma fatty acid composition was determined according to the Folch’s method [2]. Fatty acid methyl esters were analysed using a gas chromatograph (Shimadzu-2000, Japan) equipped with a 100 m column. Supelco FAME 37 (Sigma‒Aldrich, United States) was used as the standard for identifying the composition of fatty acids (mol %). A list of gastrointestinal discomfort symptoms was assessed and defined as none, mild, moderate, severe, or very severe [3]. Patients with symptoms were referred to a physician, while compliance was monitored by counting remaining softgels and recording reasons for missed doses.

**Table S4**. Outcomes with tools, kits or calculations with respective units.

| **Parameters** | **Tools/Kits/Calculations** | **Units** |
| --- | --- | --- |
| *Glycaemic markers* |  |  |
| FBG | AU680 Clinical Chemistry Analyser (Beckman Coulter) | mmol/L |
| HbA1c | Alere Afinion AS10 (Fisher Healthcare) | Percentage, % |
| FSI | Human INS (Insulin) Elisa Kit (E-EL-H2665) (Elabscience^®^) | microIU ml-1 |
| HOMA-IR | HOMA-IR: glucose (mmol/L) x insulin (mU/L) divided by 22.5 | N/A |
| *Blood pressures* |  |  |
| SBP | Standard digital automatic blood pressure monitor  (Omron HEM-7121, Kyoto, Japan) | mmHg |
| DBP |  |  |
| *Lipid profiles* |  |  |
| TC | AU680 Clinical Chemistry Analyser (Beckman Coulter) | mmol/L |
| HDL-C |  |  |
| LDL-C |  |  |
| TG |  |  |
| *Acceptance, safety and tolerability* | Gastrointestinal discomfort list | Self-perceived defined as none, mild, moderate, severe, and very severe based on symptoms (bloating, abdominal rumbling, flatulence, abdominal pain, nausea, vomiting, heart burn, loss of appetite, diarrhoea, and constipation) |

FBG: fasting blood glucose; HbA1c: glycated haemoglobin A1c; FSI: fasting serum insulin; HOMA-IR: homeostatic model assessment of insulin resistance; SBP: systolic blood pressure; DBP: diastolic blood pressure; TC: total cholesterol; LDL-C: low-density lipoprotein cholesterol; HDL-C: high-density lipoprotein cholesterol; TG: triglyceride

***Statistical analysis***

Data analysis of the completers was also performed. The differences between the SIO and placebo groups were assessed using independent Student’s t tests, paired t tests and chi-square tests. Longitudinal changes were examined using repeated measures analysis of covariance. For multiple comparisons in post hoc analyses, Bonferroni correction was selected where appropriate. The covariates applied in the analysis were age, physical activity frequency, smoking status and prescribed medication. The statistical Package for the Social Sciences (SPSS Inc., Chicago, IL, United States) software version 26.0 was used in analysis.

**References**

- - - 1. Akhtar S, Shetty R, Das A, *et al*. Current status of management, control, complications and psychosocial aspects of patients with diabetes in India: Results from the DiabCare India 2011 Study. *Indian J Endocr Metab* 2014; 18(3): 370.
      2. Folch J, Lees M, Sloane SG. A simple method for the isolation and purification of total lipides from animal tissues. *J Biol Chem* 1957; 226(1): 497-509.
      3. Quan C, Talley NJ, Cross S, *et al*. Development and validation of the Diabetes Bowel Symptom Questionnaire: Diabetes Bowel Symptom Questionnaire. *Alimentary Pharmacology & Therapeutics* 2003; 17(9): 1179–87.
