# Supplementary material for: Effects of Sacha Inchi (Plukenetia volubilis L.) Oil Supplementation on Hyperglycaemia, Hypertension and Hyperlipidaemia (3Hs) Patients: A Preliminary Human Trial
Source: Plant Foods Hum Nutr. 2025 Feb 25;80(1):80. doi: 10.1007/s11130-025-01309-8 (PMC11861005; doi:10.1007/s11130-025-01309-8)
Supplement: Supplementary file 2 — Supplementary Material 2 [file 11130_2025_1309_MOESM2_ESM.docx]

**Document Title: Supplementary Materials (Baseline Characteristics Of The Study Population)**

**Journal: Plant Foods for Human Nutrition**

**Article Title: Effects of Sacha Inchi (Plukenetia volubilis L.) oil supplementation on hyperglycaemia, hypertension and hyperlipidaemia (3Hs) patients: A preliminary human trial**

Nur Anis Raihana Mhd Rodzi, PhD^1^, Mastura Mohd Sopian, MMed^2^, Lai Kuan Lee, PhD^1*^

**^1^**Food Technology Program, School of Industrial Technology, Universiti Sains Malaysia, 11800 Gelugor, Pulau Pinang, Malaysia.

**^2^**Clinical Medicine Department, Universiti Sains Malaysia Bertam Medical Centre, 13200 Kepala Batas, Pulau Pinang, Malaysia.

Corresponding author*

Lai Kuan Lee, PhD

Food Technology Program, School of Industrial Technology, Universiti Sains Malaysia, 11800 Gelugor, Pulau Pinang, Malaysia

Email: [l.k.lee@usm.my](mailto:l.k.lee@usm.my)

Phone: +604-6536360

Fax: +604-6536375

**Table S5.** Baseline characteristics of the study population

| **Variable** | **SIO (n = 27)** | **Placebo (n = 27)** | **P-value** |
| --- | --- | --- | --- |
| Gender^b^ |  |  | 0.633 |
| Female | 13 (48.1) | 15 (55.5) |  |
| Male | 14 (51.9) | 12 (44.5) |  |
| Age (years)^a^ | 62.3 ± 6.4 | 63.5 ± 6.0 | 0.456 |
| Duration of 3Hs (year)^a^ | 6.5 ± 3.9 | 5.2 ± 3.3 | 0.332 |
| Medical prescription^b^ |  |  |  |
| Diabetes |  |  |  |
| Metformin | 17 (37.0%) | 9 (40.9%) |  |
| Gliclazide | 11 (23.9%) | 4 (18.2%) |  |
| Insulin Glargine | 7 (15.2%) | 3 (13.6%) |  |
| Sitagliptin phosphate | 5 (10.9%) | 2 (9.1%) |  |
| Insulin Aspart | 4 (8.7%) | 2 (9.1%) |  |
| Semaglutide | 2 (4.3%) | 2 (9.1%) |  |
| Hypertension |  |  |  |
| Amlodipine Besylate | 10 (25.0%) | 7 (29.2%) |  |
| Perindopril | 6 (15.0%) | 4 (16.7%) |  |
| Losartan | 6 (15.0%) | 1 (4.2%) |  |
| Valsartan | 4 (10.0%) | 4 (16.7%) |  |
| Telmisartan | 3 (7.5%) | 1 (4.2%) |  |
| Bisoprolol | 3 (7.5%) | 3 (12.5%) |  |
| Atenolol | 2 (5.0%) | 1 (4.2%) |  |
| Hydrochlorothiazide | 2 (5.0%) | 3 (12.5%) |  |
| Felodipine | 2 (5.0%) | 0 (0.0%) |  |
| Frusemide | 2 (5.0%) | 0 (0.0%) |  |
| Hyperlipidaemia |  |  |  |
| Atorvastatin | 16 (59.3%) | 9 (56.3%) |  |
| Simvastatin | 6 (22.2%) | 1 (6.3%) |  |
| Ezetimibe | 5 (18.5%) | 6 (37.5%) |  |
| Others |  |  |  |
| Acetylsalicylic acid (aspirin) & Glycine | 10 (50.0%) | 3 (37.5%) |  |
| Ivabradine | 6 (30.0%) | 3 (37.5%) |  |
| Trimetazidine | 4 (20.0%) | 2 (25.0%) |  |
| Smoking^b^ |  |  | 0.037* |
| Yes | 0 (0) | 6 (13.0) |  |
| No | 27 (100.0) | 21 (77.0) |  |
| Physical activity^b^ |  |  | <0.0001** |
| Min. 3x per week | 23 (85.2) | 16 (59.3) |  |
| No | 4 (14.8) | 11 (40.7) |  |
| Nutritional status^a^ |  |  |  |
| BMI (kg/m^2^) | 29.0 ± 6.0 | 28.0 ± 2.8 | 0.410 |
| WC (cm) | 98.7 ± 12.6 | 97.7 ± 4.0 | 0.681 |
| HC (cm) | 101.5 ± 10.6 | 103.2 ± 6.6 | 0.465 |
| WHR | 1.0 ± 0.07 | 1.0 ± 0.06 | 0.199 |
| LA (%) | 18.3 ± 1.6 | 17.9 ± 2.4 | 0.566 |
| ALA (%) | 0.4 ± 0.1 | 0.4 ± 0.2 | 0.862 |
| OA (%) | 9.3 ± 1.4 | 9.6 ± 0.8 | 0.593 |
| Total Omega-3 PUFAs | 9.8 ± 1.8 | 9.4 ± 2.1 | 0.694 |
| Total Omega-6 PUFAs | 30.6 ± 2.4 | 30.1 ± 1.7 | 0.337 |

^a^Results are presented as mean ± standard deviation (SD).

^b^Values are presented as n (%).

The levels of omega-3 polyunsaturated fatty acids are expressed as percentages of total plasma lipids.

**p*<0.05, ***p*<0.0001.

ALA = alpha linolenic acid; BMI = body mass index; LA = linoleic acid; WC = waist circumference; HC = hip circumference; PUFAs = polyunsaturated fatty acids; WHR = waist-to-hip ratio
